# Drivers and social implications of Artificial Intelligence adoption in healthcare during the COVID-19 pandemic

**DOI:** 10.1371/journal.pone.0259928

**Published:** 2021-11-22

**Authors:** Darius-Aurel Frank, Christian T. Elbæk, Caroline Kjær Børsting, Panagiotis Mitkidis, Tobias Otterbring, Sylvie Borau

**Affiliations:** 1 Department of Management, Aarhus BSS, Aarhus University, Aarhus, Denmark; 2 Social Science Research Institute, Center for Advanced Hindsight, Duke University, Durham, NC, United States of America; 3 Department of Management, University of Agder, Kristiansand, Norway; 4 Institute of Retail Economics, Stockholm, Sweden; 5 Department of Marketing, TBS Business School, Toulouse, France; University of Pisa, ITALY

## Abstract

The COVID-19 pandemic continues to impact people worldwide–steadily depleting scarce resources in healthcare. Medical Artificial Intelligence (AI) promises a much-needed relief but only if the technology gets adopted at scale. The present research investigates people’s intention to adopt medical AI as well as the drivers of this adoption in a representative study of two European countries (Denmark and France, *N* = 1068) during the initial phase of the COVID-19 pandemic. Results reveal AI aversion; only 1 of 10 individuals choose medical AI over human physicians in a hypothetical triage-phase of COVID-19 pre-hospital entrance. Key predictors of medical AI adoption are people’s trust in medical AI and, to a lesser extent, the trait of open-mindedness. More importantly, our results reveal that mistrust and perceived uniqueness neglect from human physicians, as well as a lack of social belonging significantly increase people’s medical AI adoption. These results suggest that for medical AI to be widely adopted, people may need to express less confidence in human physicians and to even feel disconnected from humanity. We discuss the social implications of these findings and propose that successful medical AI adoption policy should focus on trust building measures–without eroding trust in human physicians.

## Introduction

The coronavirus pandemic continues to shake the world. To this date, more than 160 million people have contracted COVID-19 worldwide, and more than 3.5 million people have lost their lives from being infected with the virus [1]. As nations around the globe are racing to contain the pandemic, one of the biggest threats is the mental and physical exhaustion of health professionals and medical resources [2, 3]. Here, new technology is believed to be key to providing a much-needed relief [4–6]. In particular, medical Artificial Intelligence (AI), enabled by the advancements in big data, computing power, and cloud storage of recent years [7], promises improvements to the speed, accuracy, and productivity of medical diagnoses and treatments used in fighting the pandemic [8]. If adopted at scale, medical AI has the potential to help break the chains of infections, administer safe diagnosis, and thus relieve the strain on medical staff and test laboratories worldwide. For example, medical AI that has been trained to diagnose COVID-19 infections through recordings of people’s coughing could be adopted as an app on modern smartphones [9]. Above all, the use of medical AI could prove a less disruptive yet as effective intervention as many currently used restrictive measures, such as nationwide shutdowns [10].

### Barriers to medical AI adoption

While the prospects of medical AI to fundamentally improve access to and delivery of health services during the pandemic are promising, research on AI adoption also highlights challenges and barriers that could prevent widespread adoption [11, 12]. For example, one study shows that people’s reluctance towards medical AI relative to human physicians is linked to the concern that the unique characteristics, circumstances, and symptoms of their situations are neglected–a phenomenon coined *uniqueness neglect* [13]. Other studies show that patients’ reluctance towards medical AI goes so far that medical AI is trusted less than a physician [13–15] even when the AI suggests patients’ desired treatments and performs at the same level of performance as its human counterpart [16].

While these findings corroborate with the systematic human aversion to algorithmic advice [17, 18], studies by Logg et al. [19] and Castelo et al. [14] suggest that lay people appreciate algorithmic advice in medical scenarios if those are objective in nature. This is generalized by research showing that AI algorithms are preferred over humans in decisions in which utilitarian goals are activated [20], suggesting the compensatory nature of medical AI adoption, and that “people may prefer algorithmic to human judgment […] when interacting with the provider might endanger his or her life (i.e., triage services for COVID-19)” [21, p. 447]. In addition, several studies suggest that the adoption of AI agents depends on psychological constructs, such as consumer trust [22–24], and personality traits, such as open-mindedness [25]; yet, mistrust in medical AI has never been tested in the context of a global health pandemic, nor the potential role of other drivers, such as mistrust in human physicians, and perceived uniqueness neglect from human physicians. Indeed, people who experience various forms of marginalization may develop mistrust towards other humans’ decisions [26], and may even increase their interactions with artificial products that mimic human intelligence [27]. Following this rationale, people who mistrust human physicians might perceive AI physicians to be more trustworthy and might be more likely to adopt medical AI. This would be in line with Turkle ‘s [28] prediction: if we expect more from technology, we might expect less from each other, and the other way around.

In sum, prior research on people’s adoption of AI in the medical domain is not only inconclusive, but it certainly does not speak to the specific circumstances surrounding the current pandemic, and it only focuses on a narrow set of variables. This poses the question of whether medical AI could be successful in working alongside human physicians as a means of easing the pandemic strain, and the social costs of medical AI adoption. To address this overarching question, the present research aims to examine people’s potential adoption of medical AI through the perspective of COVID-19 and future global health threats. In doing so, we strive to inform researchers and policy makers by fostering a productive discussion on the factors that may help drive the adoption and use of medical AI.

## Method

Two cross-sectional representative surveys of the population of Denmark and France were conducted during the first wave of the global pandemic, between April and May 2020, as part of a global research collaboration on the use of social and behavioral science to support COVID-19 responses [29]. The project received ethical approval by the Institutional Review Board at University of Kent (ID 202015872211976468), and informed written consent was obtained from all participants prior to their voluntary participation in the surveys.

### Participants

One thousand one hundred and sixty-eight individuals (age: *M* = 47.47, SD = 16.95; 50.34% females), representative of Denmark (*n* = 566; age: *M* = 48.69, SD = 17.54; 49.5% females) and France (*n* = 602; age: *M* = 46.31, SD = 16.29; 51.0% females) in terms of age and sex, participated in our study in exchange for monetary compensation.

### Procedure

The surveys were administered in the local languages of the two target countries (Danish for Denmark, French for France). Accordingly, all survey items were translated into the two local languages from English, and then back-translated, following the best practices for validation [30]. Participants answered identical questionnaires, beginning with items on social and moral psychological constructs and ending with questions on participants’ demographic background.

### Measures

Five items, presented in a randomized order, were used to measure participants’ intention to adopt medical AI (vs. human) during the COVID-19 pandemic. Among those, participants were asked to choose between being diagnosed by a human physician or a medical AI (single choice). Participants also indicated their perceived trust in a human physician (“How much would you trust the recommendation of the human provider (i.e., a physician)”; 7-point scale: 1 = absolutely not, 7 = absolutely), perceived trust in a medical AI (“How much would you trust the recommendation of the automated provider (i.e., a computer/algorithm/AI).”; 7-point scale: 1 = absolutely not, 7 = absolutely), perceived uniqueness neglect from a human physician (“And how concerned would you be that when analyzing your test results, the human provider (i.e., a physician) would not recognize the uniqueness of your condition:?”; 7-point scale: 1 = not at all concerned, 7 = extremely concerned; adapted from Longoni et al. [13]) and perceived uniqueness neglect from a medical AI (“And how concerned would you be that when analyzing your test results, the automated provider (i.e., a computer/algorithm) would not recognize the uniqueness of your condition:”; 7-point scale: 1 = not at all concerned, 7 = a great deal; adapted from Longoni et al. [13]).

In addition to the primary measures, participants answered eight standardized scales about their anti-COVID-19 policy support, belief in conspiracy theories, open-mindedness (adapted from Fujita et al. [31]), trait optimism [32], social belonging [33], self-esteem [34], risk perception, and political ideology, with some of the items developed for the sole purpose of the current project. Finally, participants indicated basic demographic information, including age, sex, socioeconomic status, and marital status. We controlled for people’s belief in conspiracy theories, as misinformation has created controversies that link COVID-19 infections to innovative technology [35, 36].

### Processing

Prior to conducting the analyses, data from both countries were merged to form a single dataset. Two variables were added in this step: a dummy variable to identify the specific country (Denmark = 0, France = 1) and a participant ID. Next, participants who failed the attention checks were excluded from the dataset (*n* = 185). A small amount of missing data was present in our final dataset, since participants could skip answering some questions due to privacy aspects. We did not impute missing data, but participants who failed to provide an answer (*n* = 8) were not included in our models. All multi-item scales were aggregated and averaged to form individual index variables that captured the respective constructs. All measures were standardized to allow for generalized linear regression analyses.

## Results

We focus our analyses on predicting participants’ preference for medical AI (relative to a human physician), using the reported perceived uniqueness neglect and perceived trust for each of the two agents (AI, human physician), alongside measured scales and items for personality traits, including people’s anti-COVID-19 policy support, belief in conspiracy theories, open-mindedness, optimism, social belongingness, self-esteem, COVID-19 risk perception, and political ideology, plus our demographic variables (i.e., age, sex, and socioeconomic status).

### Summary statistics

Summary statistics for perceived trust, perceived uniqueness neglect, and the choice of physician between Denmark and France are depicted in Table 1. In both countries, the human physician scores a higher mean for perceived trust and a close to identical mean for perceived uniqueness neglect relative to the medical AI. In terms of physician choice, the medical AI was chosen in less than 10 percent of the cases, so the great majority of participants preferred the human physician in performing COVID-19 related diagnoses. Nevertheless, the number of participants who chose the medical AI was sufficiently high (*n*_France_ = 54 and *n*_Denmark_ = 53) to perform the analyses presented in the following section.

**Table 1:** Summary statistics of main predictors and choice for Denmark and France.

		Denmark		France	
		Mean	SD	Mean	SD
Trust	Human	.76	.20	.85	.16
	AI	.56	.25	.63	.25
Uniqueness neglect	Human	.47	.24	.69	.23
	AI	.52	.24	.70	.25
		Count	Percentage	Count	Percentage
Physician choice	Human	512	90.46	542	90.79
	AI	54	9.54	55	9.21

Note: Responses standardized on a scale from 0 to 1.

### Main analyses

We used generalized linear regressions to test if the above-mentioned attributes predicted participants’ adoption of the medical AI relative to the human physician. Because the data were collected in two different countries, we initially conducted a generalized linear mixed effects analysis, using country as a random effect to account for possible between-factor differences in the natural clustering of the data. This analysis, however, revealed that country as a random effect did not account for any variance in the models and was therefore discarded. Instead, the country factor was added as a control variable in the final model (see Model 3). Three models were fitted in a stepwise manner, based on the Akaike Information Criterion (AIC) and estimated using Restricted Maximum Likelihood (REML). Standardized parameters for all models were obtained by fitting the models on standardized values of the data and 95% Confidence Intervals (CIs) were computed using the Wald approximation. Modelling results expressed as an odds ratio (OR)–a measure of association between an exposure and an outcome, which represents the odds that an outcome will occur given a particular exposure, compared to the odds of the outcome occurring in the absence of that exposure [37]–appear in Table 2.

**Table 2:** Summary of logistic regression models on adoption of medical AI.

	Model 1		Model 2		Model 3	
Predictors	OR	95% CI	OR	95% CI	OR	95% CI
Intercept	.03	.02 –.05	.03	.02 –.05	.03	.02 –.05
Uniqueness neglect [AI]	.75	.55–1.01	.77	.57–1.05	.76	.56–1.03
Uniqueness neglect [Human]	1.46	1.09–1.97	1.42	1.06–1.93	1.44	1.07–1.96
Trust [AI]	7.41	4.85–11.80	7.55	4.89–12.17	7.44	4.82–12.00
Trust [Human]	.31	.23 –.41	.31	.23 –.41	.31	.23 –.41
***Personality traits***						
Anti-COVID-19 policy support			.87	.67–1.14	.87	.67–1.14
Belief in conspiracy theories			.86	.65–1.13	.85	.63–1.11
Open-mindedness			1.79	1.08–3.00	1.92	1.14–3.26
Trait optimism			1.11	.82–1.52	1.09	.80–1.50
Social belonging			.61	.45 –.84	.64	.46 –.89
Self-esteem			1.14	.86–1.52	1.15	.86–1.54
COVID-19 risk perception			1.04	.82–1.33	1.00	.78–1.27
Political ideology			1.09	.86–1.38	1.12	.88–1.42
***Demographics***						
Age					.81	.63–1.03
Sex					.92	.72–1.16
Socioeconomic Status (SES)					1.00	.80–1.25
Rural residence					.83	.64–1.05
Country [France]					.97	.61–1.54
Observations	1129		1129		1129	
R^2^ Tjur	.20		.22		.23	
AIC	544.31		544.35		547.88	
BIC	569.45		609.72		638.41	

Note: OR = 1 Predictor does not affect medical AI adoption; OR > 1 Predictor is associated with higher odds of medical AI adoption; OR < 1 Predictor is associated with lower odds of medical AI adoption; 95% CI = 95% confidence interval (CI) estimate for the precision of the OR.

In Model 1, adoption of medical AI is regressed on the main predictors only. Results show that both uniqueness neglect from human physicians (OR = 1.46, 95% CI [1.09, 1.97]), mistrust in human physicians (OR = .31, 95% CI [.23, .41]), and trust in medical AI (OR = 7.41, 95% CI [4.85, 11.80]) significantly predict choice of physician type. That is, participants with higher perception of uniqueness neglect from human physicians, lower trust in human physicians, and higher trust in AI physicians are more likely to choose the medical AI.

Model 2 also includes the battery of personality traits in addition to trust and uniqueness neglect. Results show that of those, open-mindedness (OR = 1.79, 95% CI [1.08, 3.00]) and social belonging (OR = .61, 95% CI [.45, .84]) both significantly predict the adoption of medical AI, and that all the effects observed for trust and uniqueness neglect remain stable. This suggests that participants who are more open-minded and report lower perceptions of social belonging are more likely to choose the AI physician.

**Fig 1:**
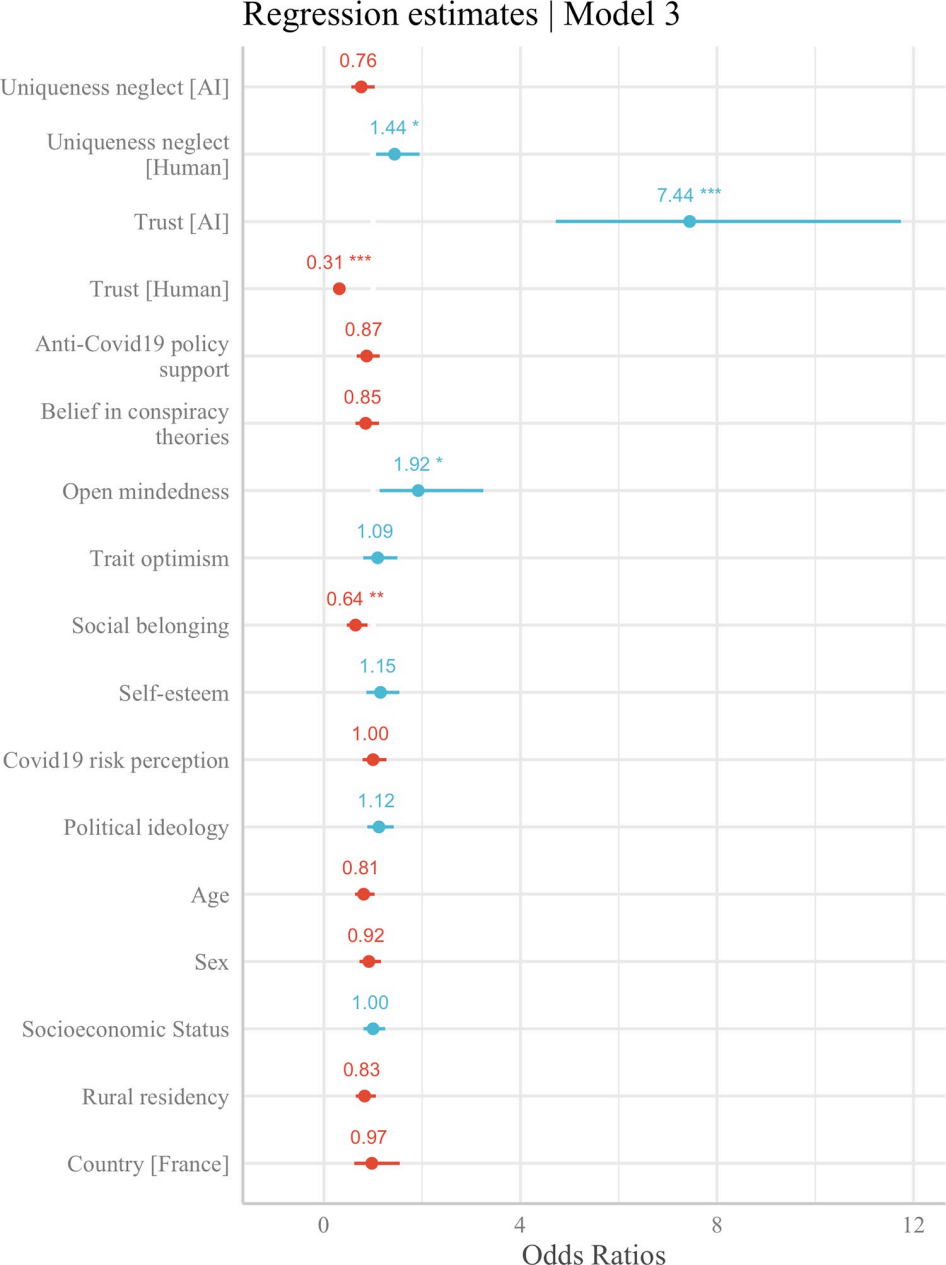
Odds ratios shown for predictors of medical AI adoption in Model 3. *Note*: Dots indicate odds ratios and lines indicate 95% confidence intervals, red (blue) indicates negative (positive) coefficients, * *p* < .05, ** *p* < .01, *** *p* < .001.

Lastly, Model 3 includes all of the previous predictors and also the basic demographics of participants. Results show that these demographic factors do not significantly predict medical AI adoption, and–once more–that the results found in Models 1 and 2 are robust to the inclusion of demographic covariates. The regression estimates for the final model (Model 3) are visualized in Fig 1.

## Discussion

To the best of our knowledge, the present research is the first to document a set of specific factors that predict people’s potential adoption of medical AI during a global pandemic. As revealed by our survey based on two representative European samples, about one in ten people would use an AI physician rather than a human physician. Our results do not corroborate the role of uniqueness neglect from AI in people’s medical AI adoption decision in the context of COVID-19. The current work does, however, reveal the roles of perceived uniqueness neglect from human physicians and people’s mistrust in human physicians, as well as their disconnection from other humans (social exclusion through a lack of social belonging) in medical AI adoption. Our findings also confirm the pivotal roles of trust in AI and open-mindedness in a health context.

### Theoretical and social contributions

The present research makes several key contributions. First, we confirm the pivotal role of trust in AI adoption [16, 23, 24, 38], particularly in the context of medical decisions [13–15], here in the context of a global health pandemic. Our results suggest that a mere one unit increase in the measure of perceived trust in the medical AI results in a seven-fold increase in the likelihood of people choosing medical AI over a human physician.

Second, previous research has shown that people’s reluctance towards medical AI is linked to the perception that their unique characteristics would not be appropriately accounted for by AI [13]. In a twist to this previous notion, we find that perceived uniqueness neglect from human physicians–not perceived uniqueness neglect from medical AI–plays a role in medical AI adoption, and, surprisingly, in a positive way. That is, people who believe that human physicians will not consider their unique characteristics are more likely to opt for an AI physician. Our findings suggest, though, that the overall magnitude of the effect of uniqueness neglect from human physicians is overshadowed by people’s trust in medical AI–probably because uniqueness neglect is an antecedent of trust in AI.

Third, our results reveal that people who score lower in perceived social belongingness are more likely to adopt medical AI. This indicates that feeling disconnected or excluded from other humans during the pandemic may push people to connect with non-human solutions. The positive effects of both lack of trust in human physicians and perceived uniqueness neglect from human physicians on adoption of medical AI corroborate this idea. The fact that technology could tear us apart and disconnect us from our humanity is widely discussed in the philosophy and ethics of AI [28, 39, 40]. However, we cannot explicitly speak to the origin of this phenomenon given the cross-sectional design of the present work. Thus, future research should investigate whether people prefer medical AI because they feel disconnected from humanity or, alternatively, whether people’s preference for AI disconnects them from humanity.

Finally, we document open-mindedness as an important personality trait to positively predict the adoption of medical AI over a human physician. Open-mindedness, therefore, appears to be a precondition for people’s adoption of such innovations, supporting evidence for the relevance of open-mindedness in AI adoption in the public sector [25], and extending it to people’s medical decisions.

### Practical and social implications

While we suggest that the widespread adoption of medical AI could help mitigate the severity of the ongoing pandemic, we acknowledge that a successful implementation strategy has to overcome strong psychological barriers in people’s beliefs about AI. Based on our findings, establishing trust in AI appears to be a precondition that is long overdue in receiving proper attention, as evidenced by the lack of trust in medical AI despite its superior properties to human physicians in certain domains, including performing specialized tasks [16]. However, increased trust in medical AI should not occur at the expense of decreased trust in human physicians. This should not be a zero-sum game as AI is meant to help and assist humans, not replace or even harm them. Consequently, managers and policy makers should strive to increase trust in medical AI without creating mistrust in medicine, science, and fellow human physicians.

Another important implication for both behavioral designers and policy makers is the lack of difference in medical AI adoption between France and Denmark. This is surprising, considering that Denmark has taken great efforts to digitize public services over the past decades, and is ranked considerably higher in terms of the public’s acceptance of technology compared to France [41]. This finding also implies that long-term policy measures, such as modernization of public services alone are insufficient to create positive responses towards innovative solutions targeted as relief measures during the ongoing pandemic. We therefore argue that policy measures aimed at facilitating necessary preconditions to the adoption of medical AI would benefit from short-term interventions that strongly focus on trust-building information campaigns in combination with incentivization.

### Limitations and future research

The main limitations of this research correspond to generalizability, as we use a representative yet ethnically homogeneous sample of European citizens [42–45], and causality, as we rely on cross-sectional data with self-report scales for explaining preferences for medical AI relative to human physicians rather than real, observable behavior [46–49]. Due to the rigorous control for confounds and reliance on domain-specific knowledge for inference of causality, we argue that our cross-sectional data can be used to ensure external validity of our findings [50]. That said, replicability of this research may be limited due to the unique background of a global pandemic. For this reason, we call for further research to validate the influence of the central constructs examined herein, to help boost trust in medical AI and AI technology in general. In the context of compensatory decision-making in AI adoption [21], it also appears interesting to investigate how much trust is generally required to meaningfully mitigate people’s reluctance towards AI technology.
